# Socio-demographic differences in the frequent use of emergency department care by older persons: a population-based study in Stockholm County

**DOI:** 10.1186/s12913-019-4029-x

**Published:** 2019-03-29

**Authors:** Megan Doheny, Janne Agerholm, Nicola Orsini, Pär Schön, Bo Burström

**Affiliations:** 1 Department of Public Health Sciences, Karolinska Institutet, Plan 6, Solnavägen 1 E, 113 65 Stockholm, Sweden; 2 Aging Research Center, Karolinska Institutet, Stockholm, Sweden; 3 Center for epidemiology and community medicine, County Council, Stockholm, Sweden

**Keywords:** Emergency department, Older people, Utilisation, Frequent users, Social differences, Inequality, Health and social care system, Ageing in place

## Abstract

**Background:**

In Sweden, the number of older people using emergency department (ED) care is rising. Among older persons an ED visit is a stressful event, which potentially could have been prevented or treated at other levels of care. Frequent ED use (> 4 visits a year) by older persons might reflect issues in the organisation of health care system to address their needs. We aimed to explore socio-demographic differences among older people seeking ED care in terms age and gender, and to investigate the association between income and frequent ED use.

**Methods:**

A population-based study analysing the utilisation of ED care by (*N* = 356,375) individuals aged 65+ years. We linked register data on socio-demographic characteristics from 2013 to health care utilisation data in 2014. Multivariable logistic regression was used to estimate the income differences in the frequent use of ED care, adjusting for living situation, country of birth, residential area, age in years, multi-morbidity and the use of other health care services.

**Results:**

Those 65+ years accounted for (27%) of all ED visits in Stockholm County in 2014. In the study population (2.5%) were identified as frequent ED users, who were predominantly in the lower income groups, living alone or in an institution, had more multi-morbidity, and utilised more of other health care services. The lowest income groups had a three-fold greater odds of being a frequent ED user than those in the highest income group. In the adjusted models, the odds were reduced by 12–44% for those in the lowest income groups. However, age and gender differences were observed with men 65–79 years (OR 1.75 CI: 1.51–2.03) and women 80+ years (OR 1.50, CI 1.19–1.87) in the lowest income groups having a higher odds of frequent ED use.

**Conclusion:**

This study observed that ED visits by older persons are driven by a need of care, and those that frequently visit hospital-based EDs are a socially disadvantaged group, which suggests that the organisation of care for older people should be reviewed in order to better meet their needs in other levels of care.

**Electronic supplementary material:**

The online version of this article (10.1186/s12913-019-4029-x) contains supplementary material, which is available to authorized users.

## Background

Older people are the fastest growing segment in the population, both in Sweden and in most other countries. In Sweden today, there is half a million people 80 years and older, which is expected to increase by 50% in 2028 [1]. Older persons have complex health problems characterised by chronic conditions, functional limitations, physical and cognitive decline [2], which require various combinations of health and social care services to meet their individual needs [3]. In Sweden, the ageing population presents a challenge for the current health and social care system, which is highly fragmented into specialised services, and is not optimally organised to address the needs of the ageing population [4]. Lately, more attention has been paid to the growing number of older people seeking emergency department (ED) care [5, 6], which may suggest that the health and social care systems are failing older persons who are the main users.

Swedish health and social care policy share an explicit equality perspective that care services should be provided according to the principle “equal access according to need”. In the Swedish context this policy refers to both horizontal equity, meaning that persons with the same medical need should have the same treatment available to them, and vertical equity which entails prioritizing persons with greater need before those with lesser needs [7, 8]. However, social differences in the use of health care services exist in Sweden, also in the older population [9–11], and remain even in the last year of life [12]. In older ages equitable distribution of health care services becomes increasingly important in order not to exacerbate the underlying inequities in health as the need for health care services increase and individuals become more dependent on health and social care to manage everyday life.

### A changing health and social care system

The Swedish health and social care system for older people has undergone drastic changes in recent decades. The prevailing policy and practice is guided by the goal of giving older people the opportunity to ‘age in place’, that is, to remain in the community rather than in institutions [13]. Since the 1992 Community Care Reform, the number of hospital beds has been reduced by over 50%. Currently, Sweden has 2.4 hospital beds per a 1000 population, this is below the OECD average of 4.7 hospital beds per a 1000 population [14]. The consequences of the decrease in hospital beds has been further intensified by a 30% reduction in municipal institutional care since 2000 [13]. The cutbacks in institutional resources have resulted in an increasing number of frail older people with complex health problems who are dependent on receiving help in their own homes from many formal and informal care sources [15].

The introduction of market-orientated reforms have influenced the provision of health and social care with increased competition through the free establishment of providers, freedom of choice and diversity [16, 17]. The Primary Care Choice Reform transformed the organisation of primary care as the provider now decides where to establish their clinic and the patients’ must choose where to receive care. In Stockholm County, prior to this reform primary care resources were distributed using a need-based allocation system, meaning that providers in areas with populations that had poorer health and greater needs received more resources than those in other areas [17]. Post reform, resources have been moving from disadvantaged areas to other areas, which only can partly be explained by area differences in the use of primary care [18]. Further, a similar choice reform in 2009 in social care has resulted in a large increase in the number of private providers of social care to older persons [19].

Hence, more resourceful individuals with a higher education, income and more social support will be better able to navigate this complicated system and to meet their needs. However, less resourceful individuals might have difficulties in navigating the system resulting in unmet needs. This may increase the risk of older persons having unmet needs and seeking hospital-based ED care.

### Increased ED visits and frequent ED use by older people

Older people have a greater tendency to visit hospital-based ED care than younger people [20], moreover, they often have more urgent health problems which drive them to seek ED care [21, 22]. However, the ED is a stressful environment that is not always an appropriate setting to address the complex health problems of older people, since they often present with diffuse symptoms, and as such, their health problems can appear less urgent, resulting in longer waiting times [23], under treatment [22], poor continuity of care, repeated ED visits due to unmet needs [24], and adverse health outcomes [25, 26].

Older people are often blamed for organisational issues in hospital-based ED’s such as increasing health care costs [27] and overcrowding [28]. However, older people’s use of ED care is often justified, and their frequent ED visits are often a consequence of persistently unmet needs that lead to health crises [20, 22, 29]. The increasing rate of ED visits by older people can be seen as an indicator of how effective community-based care is at managing chronic and acute conditions [29]. Frequent users are a vulnerable patient group, who often have complex health and social problems [30, 31], and complement their frequent ED use with heavy use of other outpatient care [32] and social care services [30]. Generally, this group of patients have a poor social network [31, 32], lower education and low income [26].

Despite frequent ED users being a well-studied group, more studies are required using population-based data to investigate the socio-demographics differences among older people seeking ED care within the context of Sweden. Previous studies investigating the population at EDs in Stockholm County, used qualitative methods and focused on appropriate use [33] and socially vulnerable groups [31] or based in one of the main hospitals in Stockholm County [23, 32, 34]. The overall aim is to study the socio-demographics differences among older people seeking ED care in terms gender and age groups, and to investigate the association between income groups and frequent ED use.

1. What are the socio-demographic characteristics of frequent ED users among older people?
2. Does adjusting for socio-demographic characteristics, multi-morbidity and utilisation of other care services weaken the association between income and frequent ED use among older persons? Are there differ by gender and age groups?

## Methods

### Study population

The study was designed as a population-based cohort study following residents 65+ years living in Stockholm County between the 1st of Jan till the 31st of Dec 2014. We excluded those who moved out of Stockholm County during follow-up (*n* = 2153), while including those who died during follow-up (*n* = 13,448), the final study population consisted of (*n* = 356,375) persons.

### Data sources

Socio-economic and demographic data was obtained from the Longitudinal Integration database for Health Insurance and Labour Market Studies (LISA) from Statistics Sweden. The LISA database is a collection of variables from different population registers. We used the LISA data from 2013 in order to have complete data for the entire population including those that died. We used the variables age, gender, and country of birth, residential area, civil status, family type and individual disposable household income.

The LISA data was linked individually through anonymised encrypted personal numbers to data from Stockholm County Council on health care utilisation and the National Board of Health and Welfare databases on cause of death. The Stockholm County Council’s administrative database for analysis and follow-up of healthcare utilisation (VAL) was used to obtain data on the utilisation of health care services during 2014. This database contains information on all registered outpatient and inpatient care visits financed by Stockholm County Council. For the purpose of this study we obtained data of all outpatient care services, including ED care, home-health care services, primary care, and specialist care and identified persons residing in institutional care.

The National Board of Health and Welfare’s Cause of Death Register contains information on the date of death and the cause of death with the corresponding ICD-10 code. For the purposes of this study we used the date of death to identify those that died in 2014.

### Variable specification

#### Outcome variable

An ED visit was defined as an outpatient visit to a hospital-based Emergency Department (ED) in Stockholm County. The utilisation of ED care was analysed as a count variable and placed into categories based on the number of visits, those with one, two to three and four or more visits. Those with four or more visits during a 1 year period were categorised as being frequent ED users [35].

#### Socio-demographic variables

Sex was categorized into men and women. Age was calculated from the registered year of birth, and divided into those 65–79 years and 80+ years, age in years was used for the regression analysis. Individual income was used as a measure of socio-economic position (SEP), grouped into categories based on the distribution of the net annual equalised individual household income and then ranked into income quintiles. Up to SEK 151,500, SEK 151,500-194,100, SEK 194,100-255,800, SEK 255,800-365,030, and greater than SEK 365,030, respectively. Country of birth was dichotomized into Sweden or outside of Sweden. Living situation was measured by combining registered civil status and family type, to distinguish those who lived with a registered partner or others from those who lived alone. For the analysis the variable living situation was placed into categories for cohabiting and living alone in the community and living in institutional care. A dichotomous division of residential areas in Stockholm County distinguished disadvantaged areas with high levels of unemployment, low levels of education and high proportion of foreign born individuals, which were identified by the Metropolitan Development Initiative in 1998 [36], from the rest of Stockholm County.

#### Health and health care utilisation variables

A baseline measure of need was applied by using a validated measure of multi-morbidity in the older population [37], compiling 60 aggregated categories of disease with corresponding ICD-10 codes, which reflects the burden of multi-morbidity among those 65+ years (see Additional file 1). We formed these categories by obtaining the registered diagnoses attached to inpatient and outpatient visits from 2011 to 2013. For the descriptive analysis multi-morbidity was grouped as having no conditions, one condition, two to four conditions and five or more conditions. Multi-morbidity was adjusted for in the regression analysis as a continuous variable (i.e. the number of chronic conditions).

Home-health care is a service provided in the patient’s home by a healthcare professional, there are two levels: basic and advanced which are provided based on medical need. Basic home-care is at least two home visits by registered nurse for a medical treatment during a month. Advanced home-health care is provided to manage the symptoms of severe chronic illnesses, providing a similar level of medical treatment to hospital-based care. For the analysis persons were identified as having received basic or advanced home-health care at any point during 2014. The utilisation of outpatient care was measured as the total number of primary care and specialist care visits with a doctor. For the analysis was categorized into less than 5 visits, 6–10 visits and >  10 visits in 2014.

### Statistical analysis

Data was analysed in SAS 9.4. The analysis was done separately for men and women, and by age groups 65–79 years and 80+ years. The composition of the study population is described in percentages in (Table 1). The outcome of interest the use of ED care is described in Table 2, in the rate of ED visits per 1-year person-time and the proportion of frequent ED users.

**Table 1:** Description of the study population in proportions (%) stratified by age group and sex

			65–79 yrs		80+ yrs	
		Total	Men	Women	Men	Women
		*N* = 356,375	*n* = 124,232	*n* = 138.187	*n* = 34,263	*n* = 59,693
		(%)	(%)	(%)	(%)	(%)
Income	Group 1 (low)	19.8	13.6	18.7	16.0	37.2
	Group 2	20.0	13.7	18.5	29.5	30.9
	Group 3	19.9	19.1	19.7	28.1	17.7
	Group 4	19.9	24.8	21.7	15.5	8.2
	Group 5 (high)	20.0	28.3	20.9	10.6	5.8
	Missing	0.4	0.5	0.4	0.3	0.2
Residential area	Rest	96.6	96.4	96.3	97.1	97.1
	Disadvantaged	3.4	3.6	3.7	2.9	2.9
Country of birth	Sweden	79.7	79.7	78.9	82.8	79.9
	Other	19.6	19.8	20.8	15.7	18.5
	Missing	0.72	0.5	0.3	1.5	1.6
Living situation	Cohabiting	47.9	62.1	47.2	51.2	17.9
	Alone	47.8	36.8	51.6	39.3	66.6
	Institution	4.4	1.1	1.2	9.5	15.5
Multi-morbidity	None	19.6	25.3	20.8	10.1	10.6
	1 condition	19.0	21.1	21.4	12.4	12.7
	2 to 4 conditions	41.5	38.9	42.8	42.4	43.6
	≥5 conditions	19.9	14.7	15.0	35.1	33.1
Home-health care	None	92.9	97	96.9	84.2	80.4
	Basic care	6.0	2.0	2.3	13.6	18.3
	Advanced care	1.1	1.0	0.8	2.2	1.3
Oupatient visits	0–5 visits	37.1	44.8	38.6	23.7	25.0
	6–10 visits	18.5	18.7	18.5	16.5	16.2
	> 10 visits	44.4	36.5	41.6	59.8	58.8
ED visits	None	74.9	79.4	80.3	60.2	61.2
	1 visit	14.7	12.6	12.4	20.2	21.1
	2–3 visits	7.9	6.1	5.6	14.3	13.5
	4+ visits	2.5	1.9	1.6	5.3	4.2
Died in 2014	Died	3.8	2.0	1.3	10.2	9.4

**Table 2:** Description of the rate of ED visits per a year of person-time and the proportion of frequent ED users

				65–79 yrs				80+ yrs			
		Totsl		Men		Women		Men		Women	
		*N* = 356,375		*n* = 124,232		*n* = 138,187		*n* = 34,263		*n* = 59,693	
		ED rate	FED%	ED rate	FED%	ED rate	FED%	ED rate	FED%	ED rate	FED%
	Total =	0.56	2.5	0.43	1.9	0.37	1.6	1.12	5.3	0.95	4.2%
Income	Group 1 low	0.77	3.6	0.65	3.4	0.54	3.6	1.32	6.4	1.00	4.4
	Group 2	0.74	3.4	0.60	2.9	0.48	2.3	1.16	6.3	0.99	4.4
	Group 3	0.55	2.5	0.44	2.0	0.35	1.4	1.08	5.2	0.86	4.1
	Group 4	0.39	1.6	0.35	1.4	0.27	1.0	0.97	5.0	0.81	3.7
	Group 5 high	0.35	1.3	0.30	1.1	0.26	1.9	1.01	4.6	0.87	2.7
Residential area	Rest	0.56	2.5	0.42	1.9	0.37	1.6	1.13	5.4	0.95	4.1
	Disadvantaged	0.55	3.2	0.51	3.0	0.46	2.3	0.73	3.6	0.85	5.9
Country of birth	Sweden	0.56	2.5	0.41	1.9	0.37	1.5	1.13	5.4	0.95	4.1
	Other	0.45	2.4	0.38	2.0	0.36	1.6	0.71	4.3	0.70	4.2
Living situation	Cohabiting	0.44	1.9	0.37	1.6	0.30	1.2	1.03	4.9	0.83	3.7
	Alone	0.63	3.0	0.49	2.4	0.42	1.9	1.18	5.8	0.98	4.5
	Institution	1.04	4.2	1.24	6.4	0.96	5.1	1.32	5.2	0.93	3.2
Multi-morbidity	None	0.20	0.5	0.17	0.4	0.15	0.4	0.48	1.5	0.45	1.0
	1 condition	0.29	0.8	0.24	0.8	0.21	0.6	0.59	2.0	0.59	1.3
	2–4 conditions	0.49	1.9	0.41	1.7	0.36	1.3	0.85	3.5	0.75	2.8
	≥5 conditions	1.31	7.4	1.16	6.7	0.96	5.7	1.82	9.8	1.50	8.2
Home-health care	None	0.44	1.7	0.37	1.5	0.32	1.2	0.86	3.4	0.73	2.4
	Basic care	1.98	12.7	2.03	16.2	1.81	12.2	2.40	14.7	1.84	11.1
	Advanced care	3.16	18.8	3.26	19.1	2.88	18.7	3.64	21.3	2.95	16.1
Outpatient care	0–5 visits	0.23	0.2	0.14	0.1	0.10	0.1	0.88	0.4	0.66	0.4
	6–10 visits	0.34	0.7	0.28	0.6	0.22	0.3	0.72	1.4	0.60	1.3
	> 10 visits	0.93	5.2	0.85	4.1	0.70	3.6	1.33	8.3	1.17	6.7
Died in 2014	Survived	0.43	2.2	0.35	1.7	0.33	1.4	0.74	4.7	0.70	3.9
	Died	3.79	9.6	4.02	11.8	3.68	11.9	4.41	11.0	3.33	7.1

*The proportion of frequent ED users (FED %)

*The rate of ED visits per a year person-time

Logistic regression models were used to estimate the income differences in the frequent use of ED care and yielded results of odds ratios (OR) with 95% confidence intervals (CI 95%). Logistic regression was chosen because of the binary outcome and both continuous and categorical independent variables included in models. The unadjusted model, showing the association between income and frequent ED use is included in Table 3. The independent variables adjusted for in models 1 and 2, were selected based on previous research [20–34]. Model 1, included age in years, living situation, residential area, country of birth and multi-morbidity. Model 2, further adjusted for variables in model 1 and the utilisation of home-health care and outpatient care visits.

**Table 3:** Logistic regression models examining the frequent ED visits and income groups, (a) 65–79 years and (b) 80+ years

	Model 0	Model 1	Model 2
	OR (95% CI)	OR (95% CI)	OR (95% CI)
(a)			
Men (65–79 yrs)			
Income group 1 low	3.13 (2.75–3.56)	2.09 (1.80–2.42)	1.75 (1.51–2.03)
Income group 2	2.67 (2.34–3.05)	1.53 (1.32–1.76)	1.37 (1.14–1.58)
Income group 3	1.77 (1.54–2.02)	1.19 (1.03–1.36)	1.11 (0.97–1.28)
Income group 4	1.26 (1.10–1.45)	1.07 (0.93–1.23)	1.00 (0.87–1.15)
Income group 5 high	1.00	1.00	1.00
Age in years		1.04 (1.03–1.05)	1.01 (1.00–1.02)
Living alone		1.30 (1.18–1.42)	1.18 (1.07–1.30)
Institutional care		1.72 (1.36–2.18)	3.46 (2.72–4.41)
Disadvantaged area		1.25 (1.04–1.51)	1.26 (1.04–1.52)
Born outside Sweden		0.91 (0.82–1.02)	1.00 (0.89–1.11)
Multi-morbidity		1.37 (1.35–1.39)	1.19 (1.17–1.20)
Basic care			3.35 (2.96–3.81)
Advanced care			5.22 (4.44–6.12)
6–10 outpatient visits			4.73 (3.46–6.47)
>10 outpatient visits			24.61 (18.77–32.26)
Women (65–79 yrs)			
Income group 1 low	2.91 (2.52–3.36)	1.74 (1.48–2.06)	1.51 (1.28–1.79)
Income group 2	2.62 (2.27–3.04)	1.54 (1.31–1.80)	1.39 (1.18–1.63)
Income group 3	1.55 (1.32–1.82)	1.11 (0.94–1.31)	1.05 (0.89–1.23)
Income group 4	1.08 (0.91–1.28)	0.96 (0.81–1.13)	0.91 (0.76–1.07)
Income group 5 high	1.00	1.00	1.00
Age in years		1.03 (1.02–1.04)	0.91 (0.76–1.07)
Living alone		1.07 (0.97–1.19)	0.99 (0.89–1.10)
Institutional care		1.53 (1.19–1.97)	3.26 (2.52–4.21)
Disadvantaged area		1.13 (0.93–1.38)	1.09 (0.89–1.34)
Born outside Sweden		0.89 (0.80–0.99)	0.93 (0.84–1.04)
Multi-morbidity		1.40 (1.38–1.41)	1.23 (1.21–1.24)
Basic care			3.18 (2.79–3.62)
Advanced care			6.82 (5.77–8.06)
6–10 outpatient visits			2.62 (1.84–3.74)
>10 outpatient visits			18.05 (13.92–24.78)
(b)			
Men (80+ yrs)			
Income group 1 low	1.42 (1.17–1.72)	1.58 (1.29–1.92)	1.54 (1.26–1.89)
Income group 2	1.17 (0.97–1.39)	1.15 (0.96–1.38)	1.11 (0.92–1.34)
Income group 3	1.16 (0.97–1.39)	1.19 (0.99–1.43)	1.14 (0.95–1.36)
Income group 4	1.09 (0.90–1.33)	1.12 (0.92–1.38)	1.10 (0.90–1.36)
Income group 5 high	1.00	1.00	1.00
Age in years		1.03 (1.02–1.05)	1.01 (1.00–1.03)
Living alone		1.11 (1.00–1.23)	1.00 (0.90–1.11)
Institutional care		0.80 (0.67–0.96)	2.12 (1.75–2.56)
Disadvantaged area		0.70 (0.49–1.00)	0.69 0.49–1.00)
Born outside Sweden		0.89 (0.77–1.02)	0.95 (0.82–1.09)
Multi-morbidity		1.25 (1.24–1.27)	1.15 (1.13–1.17)
Basic care			2.60 (2.32–2.93)
Advanced care			4.00 (3.29–4.86)
6–10 outpatient visits			3.35 (2.23–5.04)
>10 outpatient visits			14.14 (9.94–20.11)
Women (80+ yrs)			
Income group 1 low	1.66 (1.34–2.06)	1.56 (1.25–1.95)	1.50 (1.19–1.87)
Income group 2	1.67 (1.35–2.08)	1.56 (1.24–1.94)	1.47 (1.18–1.85)
Income group 3	1.52 (1.21–1.91)	1.49 (1.18–1.88)	1.40 (1.11–1.77)
Income group 4	1.39 (1.08–1.79)	1.43 (1.10–1.85)	1.37 (1.05–1.78)
Income group 5 high	1.00	1.00	1.00
Age in years		1.04 (1.03–1.05)	1.02 (1.01–1.03)
Living alone		1.04 (0.92–1.18)	0.94 (0.83–1.07)
Institutional care		0.66 (0.56–0.78)	1.82 (1.53–2.18)
Disadvantaged area		1.43 (1.15–1.77)	1.39 (1.12–1.73)
Born outside Sweden		1.08 (0.97–1.20)	1.13 (1.01–1.25)
Multi-morbidity		1.31 (1.29–1.33)	1.21 (1.20–1.23)
Basic care			2.46 (2.23–2.71)
Advanced care			3.56 (2.88–4.39)
6–10 outpatient visits			3.28 (2.37–4.55)
>10 outpatient visits			11.68 (8.82–15.48)

*OR* odds ratio and *CI* confidence interval 95%

Model 0: unadjusted

Model 1: age in years, living situation, country of birth, and multi-morbidity

Model 2: model 1, home-health care and outpatient care visits

## Results

Persons 65+ years and older accounted for 27% of all visits to hospital-based EDs in Stockholm County in 2014. In our study population (*n* = 356,375), 89,541 individuals made a total of 167,973 visits to ED care. We identified 2.5% of our study population as frequent ED users, this group accounted for 29.8% of all ED visits made by those 65+ years.

### Composition of study population

Table 1 describes composition of the study population. The majority (73.6%) were aged 65–79 years, and 55.5% were women. The distribution of income varied between age groups, with greater proportions of those aged 65–79 years having a high income (groups 4 and 5) and a greater proportion of those 80+ years in the lowest income groups (1 and 2). There were 3.4% of the study population resided in disadvantaged areas and a fifth of the population were born outside of Sweden. Living alone was the most common living situation, though more women lived alone than men. Furthermore, 4.4% of the study population were living in institutional care. Over 80% of the study population had a chronic condition. Those 80+ years received more home-health care than those aged 65–79 years, women received more basic and men received more advanced home-health care. In general, those 80+ years used more outpatient care, with 59% of men and 58% of women having more than ten visits. In 2014, 3.8% of the study population died.

### Description of emergency department utilisation

Table 2 describes the utilisation of ED care by the study population. Men had a higher rate of ED visits than women, and those 80+ years had a higher rate of ED visits than those 65–79 years. Persons in the lowest income group had a higher rate of ED visits than those in highest income group. Among all persons 65–79 years and women 80+ years, those residing in disadvantaged areas, who were born outside of Sweden and living alone had higher rates of ED visits. As the burden of multi-morbidity increases, the rate of ED visits increased across all groups. Those who received advanced home-health care had higher rates of ED visits than those who received basic home-care. Those 65–79 years in institutional care had a higher rate of ED visits than those in the community. However, women 80+ years in institutional care had a lower rate of ED visits than those living in the community.

Similar socio-demographic differences could be observed with the proportion of frequent ED users (FED in Table 2), to what was observed with the rate of ED visits. There were more frequent ED users in the lowest income groups and among those living alone, residing in disadvantaged areas and born outside of Sweden. As the burden of multi-morbidity increased, the proportion of frequent ED users increased. Frequent ED users had more visits to other outpatient care and (30.4%) of those who received home-health care services were frequent ED users. Among those in institutional care 4.2% were frequent ED users. Of those who died during follow-up 9.6% were frequent ED users.

### Adjusted odds ratios of frequent ED visits

Table 3, shows the results of unadjusted and adjusted logistic regression models of the income differentials and the factors associated with frequent ED use. In the unadjusted model, those 65–79 years in the lowest income group had more than three times higher odds of being a frequent ED user than those in the highest income group. There was a less pronounced gradient among those 80+ years with men in the lowest income group and women in groups 1 and 2 having a higher odds of being a frequent ED user.

In model 1, the association between income and the odds of being a frequent ED users was attenuated among persons in lowest income group, after adjusting for other socio-demographic factors and multi-morbidity. Among men 65–79 years all variables adjusted for in model 1 were significantly associated with an increased odds of frequent ED use, except for being born outside of Sweden. The results were similar among women 65–79 years, however, living alone was not significantly associated with increased odds of frequent ED use. Those 80+ years differed from those 65–79 years in terms of living situation, as residing in institutional care was associated with a decreased odds of being a frequent ED user. There were slight differences between men and women 80+ years, as men living alone and women residing in a disadvantaged area increased odds of being a frequent user.

In model 2, after fully adjusting for all variables including the utilisation of other health care services, the association between income and frequent ED use was further attenuated but remained significant for both genders and age groups. Among persons 65–79 years in income groups 1 and 2 had a significantly increased odds of being a frequent ED users compared to the highest income group, with men in the lowest group having a higher odds than women. While among those 80 + years, men in the lowest income group and women in all income groups (1, 2, 3 and 4) had a significantly higher odds of being a frequent ED user compared to those in the highest income group.

The utilisation of other health care services was significantly associated with frequent ED use for all groups. Consistently, those receiving home-health care either basic or advanced had a significantly increased odds of being a frequent ED user than those not receiving this form of care. Being in the last year of life was associated with an increased odds of being a frequent ED user, there was only slight differences between gender and age groups. In model 2, being born outside of Sweden was a significant predictor of frequent ED use among women 80+ years. For gender and all age groups, having had more than ten outpatient visits was associated with a substantially increased odds of being a frequent ED user, with men having a greater odds than women, and those 65–79 years having a greater odds than those 80+ years.

## Discussion

### Main findings

In this study we found that there were socio-demographic differences in the use of ED care among those 65+ years. ED users were more often in the lower income groups and residing in disadvantaged areas, those living alone or in an institution used more ED than those cohabitating. In general, those that attended ED most had increased morbidity, received basic or advanced home-health care, and had more than ten outpatient visits during follow-up. There were significant social differences in the frequent use of ED among those 65 + years, as persons in the lower income groups had a higher probability of being frequent ED users than those in the highest income group. Men 65–79 years were more likely to be frequent users than women 65–79 years, while women 80+ years were more likely to be frequent users than men. The association between income and frequent use of ED was weakened by adjusting for socio-demographic factors, multi-morbidity and the utilisation of other health care services. The remaining differences might indicate unmet need in the lower income groups. Our results highlight men 65–79 years in the lowest income groups and women 80+ years as potentially vulnerable groups.

The social gradient in the utilisation of ED care was more pronounced among those 65–79 years, which might be due to those that have a low SEP during their working life tend to experience poorer health earlier in old age [38]. Higher mortality among persons aged 65–79 years in lower socioeconomic groups may result in a less pronounced social gradient among those surviving beyond 80+ years. However, social differences do persist among those 80+ years. Our results identified women 80+ years as being a vulnerable group, as the majority lived alone, were in low income groups and had an increased odds of being a frequent ED user. This is in line with previous research that identified older women as the group most adversely affected by the cutbacks in formal care received in the home and institutional care places [39]. Living alone might indicate a sufficient level of health that enables them to manage their needs and remain living in the community. However, this increases the risk of older women experiencing an unstable living situation and difficulties in managing their care needs, which might explain why this group had an increased odds of being a frequent ED user.

### Implications of a changing health and social care system

A consequence of the marketisation of health and social care for older people has been a rapid increase in the number of providers, which has resulted in a risk of further fragmentation and challenges to providing the optimal care for older people. The heavy use of other outpatient care and the frequent use of ED care probably reflects greater morbidity, hence, a greater need of care. Among men 65–79 years and women 80+ years there was a greater proportion of frequent ED in disadvantaged areas compared to other areas, which may reflect the variation in the availability, and potentially the quality of, primary care depending on the patient’s area of residence in Stockholm. Other studies have found that a majority of adults who visited the ED considered the availability of primary care to be good [40], but that the care they received was insufficient or poor quality or a lack of trust between patient and provider [41].

Our study did not investigate how social care or the interaction between primary care and social care impacted on the frequent use of ED care. However, a recent government investigation [42] found that the primary care choice reform had been counteracting collaboration around older persons with complex health and social care needs. The multitude of providers requires older persons to make choices, which in turn requires knowledge about the services and quality of providers. The social differences observed in frequent use of ED care are possibly associated with the increased fragmentation and individualisation or the Swedish health and social care system, which greatly impacts older people who are the main users. Further studies are needed that assess these reforms from a more longitudinal perspective and that assess other performance indicators to better understand the impact of this changing health and social care system on older persons.

“Ageing in place” has become the reality for the majority of the study population that are now living alone in the community and a small proportion residing in institutional care. Many challenges have emerged from this policy, as the number of frail older persons with complex problems living in their own homes will put extraordinary demands on the primary and home-health care as well as the municipal home help systems. The reductions in the number of places in institutional care will impact the organisation of palliative care and the quality of life of older persons prior to death. Among those living in the community, receiving home-health care, and being in the last year of life were both significant predictors of being a frequent ED user. Similar results have been reported from the UK, in which 56% of older persons who attended the ED had received palliative care 3 months prior, and often had complex medical and social needs during that time [43]. Furthermore, an Australian study suggested that a gap might exist between the care provided by other outpatient care and home-health care, which results in patients frequently seeking ED care [44]. This pertains particularly to those 80+ years, as the results reflect that persons in institutional care used ED care less frequently than their counterparts in the community. The reduction in the number of institutional care places might have led to unmet care needs, with an increasing number of older people dependent on receiving care and dying in their homes.

### Unmet health and social care needs

The frequent use of ED by older people is often driven by a need of care [26, 29], which is demonstrated in our results as age and multi-morbidity were associated with being a frequent ED user. Older people have chronic condition(s) that constitute a medical need for care, and often when they visit an ED it is necessary and unavoidable at the time [22, 45]. However, the complex health problems of older people would be more appropriately treated and managed in other levels of care, since better access to primary care has been associated with reductions in the use of ED care by older persons [26, 46]. If needs were better addressed in other outpatient and social care, being older and/or having multi-morbidity should not be a determinant of ED use. This may reflect a failure of the health and social care to meet the complex needs of older people, as the optimal provision of, and access to primary and home-health care, as well as social care services should mitigate the need to frequently visit ED care.

### Strengths and limitations

The strengths of this study is the inclusion of all residents of Stockholm County and the ability to link multiple population-based registers to follow all persons 65+ years, and even the oldest-old. Using income to measure SEP allowed for more variation to be observed between social groups, it is a good indicator of standard of living and economic resources. Furthermore, income indicates previous labour market experiences, which impacts the health in old age [38].

Including the living situation is a strength, particularly the inclusion of those in institutional care who have either been excluded or not identified in previous studies [22]. Measuring utilisation of other health care services means patterns of use can be observed and allows to identify the groups accessing different types of care, which in turn, increases our understanding of the overall health care system. Finally, as death most often takes place in older ages, by including those that died in the study population we were able to analyse the use of ED care in the last year of life, which is important as a large part of the healthcare consumed in a lifetime is consumed in the end of a person’s life [12].

There are several limitations in this study, firstly, not having a measure of self-reported health status, to better understand how frequent ED users perceived their health. Secondly, we could not distinguish a frequent user from an inappropriate ED user, as we do not have the reason for the ED visit. Thirdly, in the literature there is no agreed upon measure of frequent ED use, the number of visits can vary from three to twelve over different time period. The definition used in this study was used to be comparable to previous studies focused on ED care is Stockholm [31, 32, 34]. However, this may limit the comparisons to other studies with different definitions, and between different health care systems.

Finally, the instrument used to measure multi-morbidity has limitations as it measures the count of chronic conditions, hence, does not assign different weights to conditions that require different levels of treatment and cause varying degrees of disability, or different clusters of disease. However, it is a verified measure and our results are comparable to what was found in the original study [37].

## Conclusion

This study shows that there are social and demographic differences among older persons that use ED care. It is clear that ED visits by older persons are driven by a need of care, and those that frequently visit hospital-based EDs are a more disadvantaged group, which suggests that the organisation of care for older people should be reviewed in order to better meet their needs at other levels of care. Responding to the needs of frail older people with multiple and chronic health problems living in their own homes will put extraordinary demands on the primary care as well as the home-health care and social care services to be flexible and receptive.

## Additional file

Additional file 1: Measure of multi-morbidity. Contains a comprehensive list of chronic conditions that were used to measure multi-morbidity. (PDF 95 kb)

## Abbreviations

CI: Confidence interval
ED: Emergency department
LISA: Longitudinal Integration database for Health Insurance and Labour Market Studies
OR: Odds ratio
SEP: Socio-economic position
VAL: Vardanalysis
