# Supplementary material for: Socio-demographic differences in the frequent use of emergency department care by older persons: a population-based study in Stockholm County
Source: BMC Health Serv Res. 2019 Mar 29;19:202. doi: 10.1186/s12913-019-4029-x (PMC6440084; doi:10.1186/s12913-019-4029-x)
Supplement: Supplementary file 1 — Measure of multi-morbidity. Contains a comprehensive list of chronic conditions that were used to measure multi-morbidity. (PDF 95 kb) [file 12913_2019_4029_MOESM1_ESM.pdf]

Measure of multi-morbidity

Contains a comprehensive list of chronic conditions that were included when measuring multi-morbidity.

Chronic Disease	ICD-10 Codes
Source(s) of data	Health
1. Allergy	J30.1-J30.4; J45.0; K52.2; L20; L23; L50.0; Z51.6
2. Anemia	D50-D53; D55-D59 (excl. D56.3; D59.0; D59.2; D59.3; D59.6); D60-D64 (excl. D60.1; D61.1; D61.2; D62; D64.2)
3. Asthma	J45
4. Atrial fibrillation	I48
5. Autoimmune diseases	I73.1; L10 (excl. L10.5); L12; L40; L41; L93-L95; M30-M36 (excl. M32.0; M34.2; M35.7-M35.9; M36.0; M36.1; M36.2; M36.3)
6. Blindness, visual loss	H54 (excl. H54.3); Z44.2; Z97.0
7. Blood and blood forming organ diseases	D66-D69 (excl. D68.3; D68.4; D69.5); D71; D72.0; D73.0-D73.2; D74 (excl. D74.8); D75.0; D76.1; D76.3; D77; D80 (excl. D80.7); D81-D84; D86; D89 (excl. D89.1; D89.3)
8. Bradycardias and conduction diseases	I44.1-I44.3; I45.3; I45.5; Z95.0
9. Cardiac valve diseases	I05-I08; I09.1; I09.8; I34-I38; I39.0-I39.4; Q22; Q23; Z95.2-Z95.4
10. Cataract and other lens diseases	H25-H28; Q12; Z96.1
11. Cerebrovascular disease	G45; G46; I60-I64; I67; I69
12. Chromosomal abnormalities	Q90-Q99
13. Chronic infectious diseases	A15-A19; A30; A31; A50-A53 (excl. A51); A65-A67; A69.2; A81; B20-B24; B38.1; B39.1; B40.1; B57.2-B57.5; B65; B92; B94; J65; M86.3-M86.6
14. Chronic kidney disease	I12.0; I13.0-I13.9; N01, N02, N04, N05; N07; N08; N11; N18.3-N18.9; Q60; Q61.1-Q61.9; Z90.5; Z94.0
15. Chronic liver disease	B18; K70 (excl. K70.0; K70.1); K71.3-K71.5; K71.7; K72.1; K73; K74; K75.3-K75.8; K76.1; K76.6; K76.7; K77.8; Q44.6; Z94.4
16. Chronic pancreas, biliary tract and gallbladder diseases	K80.0; K80.1; K80.2; K80.8; K81.1; K86 (excl. K86.2; K86.3; K86.9); Q44.0-Q44.5; Q45.0
17. Chronic ulcer of the skin	I83.0; I83.2; L89; L97; L98.4

18. Colitis and related diseases	K52.0; K52.8; K55.1; K55.2; K57.2-K57.5; K57.8; K57.9; K58; K59.0; K59.2; K62 (excl. K62.0; K62.1; K62.5; K62.6); K63.4; K64 (excl. K64.5);
19. COPD, emphysema, chronic bronchitis	J41-J44; J47
20. Deafness, hearing loss	H80; H90; H91.1; H91.3; H91.9; Q16; Z45.3; Z46.1; Z96.2; Z97.4
21. Dementia	F00-F03; F05.1; G30; G31
22. Depression and mood diseases	F30-F34; F38; F39; F41.2
23. Diabetes	E10; E11; E13; E14; E89.1
24. Dorsopathies	M40-M43; M47-M53; Q67.5; Q76.4; Q76.1;
25. Dyslipidemia	E78
26. Ear, nose, throat diseases	H60.4; H66.1-H66.3; H70.1; H71; H73.1; H74.1; H81.0; H83.1; H83.2; H95; J30.0; J31-J33; J34.1-J34.3; J35; J37; J38.0; J38.6; K05.1; K05.3; K07; K11.0; K11.7; Q30-Q32; Q35-Q38
27. Epilepsy	G40 (excl. G40.5)
28. Esophagus, stomach and duodenum diseases	I85; I86.4; I98.2; I98.3; K21; K22.0; K22.2; K22.4; K22.5; K22.7; K23.0; K23.1; K25.4-K25.7; K26.4-K26.7; K27.4-K27.7; K28.4-K28.7; K29.3-K29.9; K31.1-K31.5; Q39; Q40; Z90.3
29. Glaucoma	H40.1-H40.9
30. Heart failure	I11.0; I13.0; I13.2; I27; I28.0; I42; I43; I50; I51.5; I51.7; I52.8; Z94.1; Z94.3
31. Hematological neoplasms	C81-C96
32. Hypertension	I10-I15
33. Inflammatory arthropathies	M02.3; M05-M14; M45; M46.0; M46.1; M46.8; M46.9
34. Inflammatory bowel disease	K50; K51
35. Ischemic heart disease	I20-I22; I24; I25; Z95.1; Z95.5
36. Migraine and facial pain syndromes	G43; G44.0-G44.3; G44.8; G50
37. Multiple sclerosis	G35
38. Neurotic, stress-related and somatoform diseases	F40-F48 (excl. F43.0; F43.2)
39. Obesity	E66
40. Osteoarthritis and other degenerative joint diseases	M15-M19; M36.2; M36.3

41. Osteoporosis	M80-M82
42. Other cardiovascular diseases	I09 (excl. I09.1; I09.8); I28.1; I31.0; I31.1; I45.6; I49.5; I49.8; I70-I72 (excl. I70.2); I79.0; I79.1; I95.0; I95.1; I95.8; Q20; Q21; Q24-Q28; Z95.8; Z95.9
43. Other digestive diseases	K66.0; K90.0-K90.2; K91.1; K93; Q41-Q43; R15; Z90.4; Z98.0
44. Other eye diseases	H02.2-H02.5; H04 (excl. H04.3); H05 (excl. H05.0); H10.4; H17; H18.4-H18.9; H19.3; H19.8; H20.1; H21; H31.0-H31.2; H31.8; H31.9; H33; H35.2-H35.5; H35.7-H35.9; H36; H47-H49 (excl. H47.0; H47.1; H48.1); H51; Q10-Q15 (excl. Q12); Z94.7
45. Other genitourinary diseases	B90.1; N20.0; N20.2; N20.9; N21.0; N21.8; N21.9; N22; N30.1-N30.4; N31; N32.0; N32.3; N32.8; N32.9; N33; N35; N39.3; N39.4; N48.0; N48.4; N48.9; N70.1; N71.1; N73.1; N73.4; N73.6; N76.1; N76.3; N81; N88; N89.5; N90.5; N95.2; Q54; Q62.0-Q62.4; Q62.7; Q62.8; Q63.8; Q63.9; Q64.0; Q64.1; Q64.3-Q64.9; Z90.6; Z90.7; Z96.0
46. Other metabolic diseases	E20-E31 (excl. E23.1; E24.2; E24.4; E27.3; E30); E34 (excl. E34.3; E34.4); E35 (excl. E35.0); E40-E46 (excl. E44.1); E64; E70-E72; E74-E77; E79 (excl. E79.0); E80 (excl. E80.4); E83-E89 (excl. E86; E87; E88.3; E89.0; E89.1); K90.3; K90.4; K90.8; K90.9; K91.2; M83; M88; N25
47. Other musculoskeletal and joint diseases	B90.2; M21.2-M21.9; M22-M24; M25.2; M25.3; M35.7; M61; M65.2-M65.4; M70.0; M72.0; M72.2; M72.4; M75.0; M75.1; M75.3; M75.4; M79.7; M84.1; M89; M91; M93; M94; M96; M99; S38.2; S48; S58; S68; S78; S88; S98; T05; T09.6; T11.6; T13.6; T14.7; T90-T98; Q65; Q66; Q68; Q71-Q74; Q77; Q78; Q79.6; Q79.8; Q87; Z44.0; Z44.1; Z89.1-Z89.9; Z94.6; Z96.6; Z97.1
48. Other neurological diseases	B90.0; D48.2; G04.1; G09-G14 (excl. G13.0; G13.1); G24-G26 (excl. G25.1; G25.4; G25.6); G32; G37; G51-G53 (excl. G51.0); G70; G71; G72.3-72.9; G73 (excl. G73.2-G73.4); G80-G83 (excl. G83.8); G90; G91; G93.8; G93.9; G95; G99; M47.1; Q00-Q07; Q76.0
49. Other psychiatric and behavioral diseases	F04; F06; F07; F09; F10.2; F10.6; F10.7; F11.2; F11.6; F11.7; F12.2; F12.6; F12.7; F13.2; F13.6; F13.7; F14.2; F14.6; F14.7; F15.2; F15.6; F15.7; F16.2; F16.6; F16.7; F17.2; F17.6; F17.7; F18.2; F18.6; F18.7; F19.2; F19.6; F19.7; F50; F52; F60-F63; F68; F70-F89; F95; F99
50. Other respiratory diseases	B90.9; E66.2; J60-J67; J68.4; J70.1; J70.3; J70.4; J84; J92; J94.1; J95.3; J95.5; J96.1; J98 (excl. J98.1); Q33; Q34; Z90.2; Z94.2; Z94.3; Z96.3
51. Other skin diseases	L13; L28; L30.1; L43 (excl. L43.2); L50.8; L58.1; L85; Q80; Q81; Q82.1; Q82.2; Q82.9
52. Parkinson and parkinsonism	G20-G23 (excl. G21.0)

53. Peripheral neuropathy	B91; G54-G60; G62.8; G62.9; G63 (excl. G63.1); M47.2; M53.1; M54.1
54. Peripheral vascular disease	I70.2; I73 (excl. I73.1; I73.8); I79.2; I79.8
55. Prostate diseases	N40; N41.1; N41.8
56. Schizophrenia and delusional diseases	F20; F22; F24; F25; F28
57. Sleep disorders	G47; F51.0-F51.3
58. Solid neoplasms	All C (excl. C81-C96); D00-D09; D32.0; D32.1; D32.9; D33.0-D33.4; Q85
59. Thyroid disease	E00-E03 (excl. E03.5); E05; E06.2; E06.3; E06.5; E07; E35.0; E89.0
60. Venous and lymphatic diseases	I78.0; I83; I87; I89; I97.2; Q82.0
